# Oncostatin M induces RIG‐I and MDA5 expression and enhances the double‐stranded RNA response in fibroblasts

**DOI:** 10.1111/jcmm.13221

**Published:** 2017-05-30

**Authors:** Sabine Hergovits, Christine Mais, Claude Haan, Ana P. Costa‐Pereira, Heike M. Hermanns

**Affiliations:** ^1^ Medical Clinic and Policlinic II Division of Hepatology University Hospital Würzburg Würzburg Germany; ^2^ University of Luxembourg Life Sciences Research Unit‐Signal Transduction Laboratory Belvaux Luxembourg; ^3^ Department of Oncology Imperial College London London UK

**Keywords:** oncostatin M, STAT1, DExD/H‐Box RNA helicase, RIG‐I, innate immunity

## Abstract

Interleukin (IL)‐6‐type cytokines have no direct antiviral activity; nevertheless, they display immune‐modulatory functions. Oncostatin M (OSM), a member of the IL‐6 family, has recently been shown to induce a distinct number of classical interferon stimulated genes (ISG). Most of them are involved in antigen processing and presentation. However, induction of retinoic acid‐inducible gene (RIG)‐I‐like receptors (RLR) has not been investigated. Here we report that OSM has the capability to induce the expression of the DExD/H‐Box RNA helicases RIG‐I and melanoma differentiation antigen 5 (MDA5) as well as of the transcription factors interferon regulatory factor (IRF)1, IRF7 and IRF9 in primary fibroblasts. Induction of the helicases depends on tyrosine as well as serine phosphorylation of STAT1. Moreover, we could show that the OSM‐induced STAT1 phosphorylation is predominantly counter‐regulated by a strong STAT3‐dependent SOCS3 induction, as *Stat3* as well as *Socs3* knock‐down results in an enhanced and prolonged helicase and IRF expression. Other factors involved in regulation of STAT1 or IRF1 activity, like protein tyrosine phosphatase, non‐receptor type 2 (PTPN2), promyelocytic leukaemia protein (PML) or small ubiquitin‐related modifier 1 (SUMO1), play a minor role in OSM‐mediated induction of RLR. Remarkably, OSM and interferon‐γ (IFN‐γ) synergize to mediate transcription of RLR and pre‐treatment of fibroblasts with OSM fosters the type I interferon production in response to a subsequent encounter with double‐stranded RNA. Together, these findings suggest that the OSM‐induced JAK/STAT1 signalling is implicated in virus protection of non‐professional immune cells and may cooperate with interferons to enhance RLR expression in these cells.

## Introduction

Activation of pattern recognition receptors (PRR) is the first line of defence against infection by pathogens. PRR can be classified into four main families: the Toll‐like receptors (TLR), the C‐type lectin receptors (CTL), the retinoic acid‐inducible gene (RIG)‐I‐like receptors (RLR) and nucleotide binding leucine‐rich repeat (NLR) containing receptors, also known as NOD‐like receptors. The first two are membrane‐bound receptors, whereas the latter two are localized in the cytoplasm.

Many studies reveal that the RLR play an important role in the antiviral response of non‐professional immune cells, including fibroblasts, keratinocytes, endothelial cells and epithelial cells 1, 2, 3, 4, 5. While resting cells show relatively low levels of RLR, their expression is strongly induced after viral infection and exposure to type I interferons (IFN) 4, 6. It is further reported that their expression can be induced by lipopolysaccharide (LPS) 5, 7 and several inflammatory cytokines like IFN‐γ 8, 9, 10, 11, TNF‐α 4, 9, 12 or IL‐1β 13. Although IL‐6‐type cytokines have no direct antiviral activity 14, 15, several publications imply a modulatory function. IL‐6‐ or IL‐6R‐deficient mice, for example, display markedly reduced antiviral responses 16, 17.

Recently OSM, another member of the IL‐6‐type cytokine family, was shown to induce a distinct number of classical interferon target genes (ISG) 18. In combination with type I IFNs, OSM synergized in the inhibition of HCV and HAV infection in hepatoma cells by an enhanced expression of a subset of ISG as well as induction of immune stimulatory molecules like IL‐7 and IL‐15R, which are implicated in T‐cell effector functions 19, 20, 21. OSM is produced by activated monocytes and macrophages 22, 23, but can also be released by neutrophils and dendritic cells upon stimulation with LPS or granulocyte/macrophage‐colony stimulating factor (GM‐CSF) 20, 24, 25, 26, 27, 28. In the human system, OSM can bind to two receptor complexes. The type I receptor complex consists of the common signal transducer glycoprotein 130 (gp130) and the leukaemia inhibitory factor receptor (LIFRβ), and the type II receptor complex consists of gp130 and the specific OSM receptor (OSMRβ) 29, 30, 31, 32. As tissue‐resident cells, like fibroblasts, express both LIFRβ and OSMRβ, they are potential target cells of OSM. Fibroblasts are found in all tissues and referred to as sentinel cells of the connective tissue. Although fibroblasts are non‐professional immune cells, they play an important role in the innate and adaptive immunity, particularly in the early innate immune response due to their ability to produce high levels of IFN 33.

In the current study, we examined the expression of ISG upon stimulation with OSM and found that RLR as well as several IRF were strongly induced in primary human dermal fibroblasts (HDF). The expression of the pattern recognition receptors of the RLR subtype RIG‐I and MDA5 as well as of the interferon‐regulated transcription factors IRF1, IRF7 and IRF9 was rapidly induced by the OSM‐activated STAT1 within these cells. Moreover, we provide evidence that the STAT1 phosphorylation and ISG expression in HDF is predominantly counter‐regulated by a STAT3‐dependent SOCS3 expression, as knock‐down of *Stat3* or *Socs3* prolonged the OSM‐induced STAT1 signalling and therefore also helicase and IRF expression. Previous studies suggested that OSM and IFN synergize in induction of antiviral defence mechanisms in hepatocytes. Here, we show that the OSM‐induced JAK/STAT1 signalling sensitizes HDF to the encounter of double‐stranded RNA and supports IFN‐γ signalling to increase the RIG‐I and MDA5 expression.

## Methods

### Cell culture, reagents and recombinant cytokines

Human dermal fibroblasts (HDF), lung fibroblasts (HLF) and osteosarcoma cells (U2OS) were grown in DMEM. Human hepatoma cells (HepG2) were grown in DMEM‐F12. All media were purchased from Invitrogen (Darmstadt, Germany) and supplemented with 10% FCS (PAA, Cölbe, Germany). All cells were cultured at 37°C under 5% CO_2_ in a water‐saturated atmosphere. Recombinant human OSM (hOSM), human IFN‐γ (hIFN‐γ) and human IL‐6 (hIL‐6) were purchased from Peprotech (Hamburg, Germany) and recombinant human LIF from Sigma‐Aldrich (Taufkirchen, Germany). Soluble human IL‐6Rα (sIL‐6R) was generated as described 34 and kindly provided by Prof. Dr. G. Müller‐Newen (RWTH Aachen University Hospital). LPS was obtained from Cell Concepts (Umkirch, Germany). U0126 and SB202190 were obtained from Merck Millipore (Darmstadt, Germany). The double‐stranded polyribonucleotide poly(I)·poly(C) (poly I:C) was obtained from GE Healthcare (Amersham, United Kingdom).

### Cell lysis and Western Blot

Cells were stimulated either with 20 ng/ml hOSM, 1000 U/ml hIFN‐γ, 10 ng/ml hLIF, 20 ng/ml hIL‐6 together with 500 ng/ml sIL‐6R or 100 ng/ml LPS. Cells were lysed either in ice‐cold Triton X‐100 lysis buffer (20 mM Tris, pH 7.5, 150 mM NaCl, 1% Triton X‐100, 10 mM NaF, 1 mM Na_3_VO_4_, 1 mM phenylmethylsulfonyl fluoride, 5 μg/ml aprotinin, 5 μg/ml leupeptin) or 1× Laemmli buffer as described previously 35. Proteins were separated by 10% SDS‐PAGE, followed by semi‐dry Western blotting onto a PVDF membrane (Whatman, GE Healthcare). Protein detection was conducted using the indicated antibodies and the enhanced chemiluminescence kit (Thermo Fisher Scientific Inc., Darmstadt, Germany) according to the manufacturer's instructions. Equal loading of the gel was verified by stripping the membrane in 62.5 mM Tris‐HCl (pH 6.7) containing 2% SDS and 100 mM β‐mercaptoethanol at 70°C for 20 min. and redetection with antibodies recognizing the protein irrespective of its phosphorylation status as well as by detection of α‐tubulin.

### Antibodies

STAT1, STAT3, pY701‐STAT1, pS727‐STAT1, pY705‐STAT3, pT222‐MK2, MK2, pTpY‐p42/44 (ERK1/2), p42/44 (ERK1/2), IRF1 and MDA5 antibodies were purchased from Cell Signalling Technology (New England Biolabs, Frankfurt a.M., Germany); RIG‐I antibodies were obtained from Cell Signalling Technology or ProSci (Poway, CA, USA); α‐tubulin and TC‐PTP antibodies were purchased from Sigma‐Aldrich.

### Small interfering RNA (siRNA) transfection

Human *LIFR*,* STAT1*,* STAT3*,* SOCS3*,* PML*,* PTPN2* and *SUMO1* siRNA (ON‐TARGET plus SMARTpool) were purchased from Dharmacon (Thermo Fisher Scientific Inc.) and non‐silencing control siRNA (AllStars negative Control siRNA) from Qiagen (Hilden, Germany). HDF were transfected 4 hrs after plating (0.4 × 10^5^ cells per well in 12‐well plates) with 33 nM siRNA using Lipofectamine RNAiMax (Invitrogen) according to the manufacturer's instructions. At day two post‐transfection, cells were stimulated and lysed for protein detection by Western Blot analysis or RNA extraction for quantitative real‐time RT‐PCR.

### Quantitative and semi‐quantitative RT‐PCR

RNA was isolated using the RNeasy kit (Qiagen) according to the manufacturer's instructions. For quantitative RT‐PCR, up to 1 μg total RNA was used for cDNA synthesis using the Transcriptor First‐Strand cDNA Synthesis Kit from Roche Diagnostics (Mannheim, Germany). Quantitative real‐time RT‐PCR was performed using the FastStart Universal SYBR Green Master (Rox) Kit (Roche Diagnostics) according to manufacturer's instructions with specific primer pairs designed using the Universal ProbeLibrary Software (Roche) (Table 1). For each target mRNA analysed, 5 μl of FastStart Universal SYBR Green master mix, 400 nM of each primer pair, 2.2 μl nuclease‐free water and 2 μl of cDNA were mixed in 384‐well plates in duplicates using a robot (Qiagility; Qiagen), and qPCR was carried out on an ABI PRISM 7900HT (Applied Biosystems, Darmstadt, Germany): initial activation 10 min. at 95°C, 40 cycles denaturation, 15 sec. 95°C, hybridization 20 sec. 60°C, elongation 20 sec. 60°C; final elongation 15 sec. 65°C; final melting curve 15 sec. 95°C, 15 sec. 60°C. Quantification of target messages was performed as described by Pfaffl 36 using GAPDH as internal control.

**Table 1:** Primer sequences for quantitative real‐time PCR

Primer	Sequence (5′ → 3′)
hGAPDH for hGAPDH rev	AGCCACATCGCTCAGACAC GCCCAATACGACCAAATCC
hRIG‐I for hRIG‐I rev	CTTTTTCTCAAGTTCCTGTTGGA TCCCAACTTTCAATGGCTTC
hMDA5 for hMDA5 rev	GGCACCATGGGAAGTGATT GATGATGATATTCTTCCCTTCCA
hIRF1 for hIRF1 rev	CAGCCCAAGAAAGGTCCTC TTGAACGGTACAGACAGAGCA
hIRF7 for hIRF7 rev	AGCTGTGCTGGCGAGAAG CATGTGTGTGTGCCAGGAA
hIRF9 for hIRF9 rev	AGCCTGGACAGCAACTCAG GAAACTGCCCACTCTCCAC
hSTAT3 for hSTAT3 rev	CCCTTGGATTGAGAGTCAAGA AAGCGGCTATACTGCTGGTC
hSOCS3 for hSOCS3 rev	AGACTTCGATTCGGGACCA AACTTGCTGTGGGTGACCA
hSOCS1 for hSOCS1 rev	CCCCTGGTTGTTGTAGCAG GTAGGAGGTGCGAGTTCAGG
hLIFR for hLIFR rev	GGCCCGGAGAAGAGTATGTA TCACCACTCCAACAATGACAG
hPML for hPML rev	ACCTCAAGATTGACAATGAAACC ACACGGCCTTGGAGTAGATG
hSUMO1 for hSUMO1 rev	AAGCCACCGTCATCATGTCT TTATCCCCCAAGTCCTCAGTT

For semiquantitative RT‐PCR, 1 μg total RNA was reverse‐transcribed using the One‐Step RT‐PCR kit (Qiagen) and the following PCR conditions: initial activation 15 min. at 95°C, 35 cycles denaturation, 40 sec. 94°C, hybridization 30 sec. 56°C, elongation 30 sec. 72°C and final elongation 10 min. 72°C. PCR products were separated on 1% agarose gels and stained with ethidium bromide.

### ELISA

Subconfluent cultures (70–80%) were left untreated or pre‐incubated for 2 hrs with 20 ng/ml OSM before stimulation with 5 or 100 μg/ml poly I:C for 2 hrs. Supernatants were collected, concentrated and analysed by ELISA. The human IFN‐β ELISA kit was purchased from PBL Assay Science, Piscataway, NJ, USA (# 41410) and used according to the manufacturer's protocol.

### Statistical analysis

All data are given as mean + S.E.M. (standard error of means) using an unpaired, two‐tailed Student's *t*‐test or Mann–Whitney *U*‐test, unless indicated differently in the respective figure legend. A value of *P* < 0.05 (*), *P* ≤ 0.01 (**) or *P* ≤ 0.001 (***) was considered statistically significant.

## Results

### RIG‐I and MDA5 are early target genes of OSM in primary human fibroblasts

**Figure 1:**
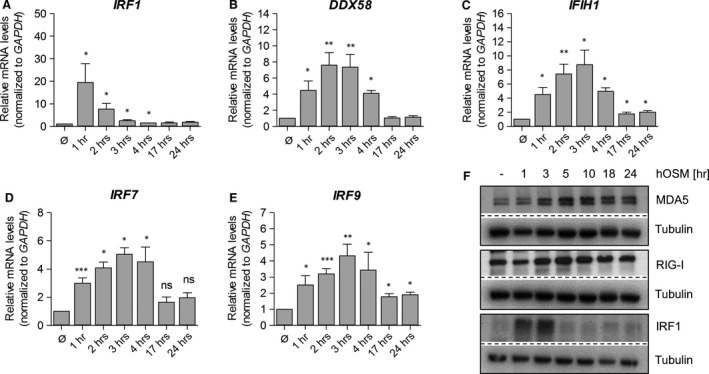
OSM induces DExD/H‐Box RNA helicase and IRF expression in human dermal fibroblasts (HDF). HDF were treated with OSM (20 ng/ml) for indicated time periods. Total RNA was prepared and subjected to reverse transcription. **(A)** *IRF1*,** (B)** *DDX58*,** (C)** *IFIH1*,** (D)** *IRF7* and **(E)** *IRF9* mRNA levels were analysed by quantitative real‐time RT‐PCR. Relative mRNA levels were normalized to the housekeeping gene, GAPDH, and fold changes were calculated relative to untreated sample (set to 1). Shown are the means (*n* = 4–5) with standard error of mean (S.E.M.). Statistical significance was assessed by unpaired *t*‐test. *P ≤ 0.05, **P ≤ 0.01, ***P ≤ 0.001 when compared to untreated sample. **(F)** Whole cellular lysates were prepared after stimulation with OSM (20 ng/ml) for the indicated time periods and expression of MDA5, RIG‐I and IRF1 was determined by Western blot analysis using specific antibodies. Tubulin was included as loading control. Blots shown are representative for three experiments.

A preliminary microarray‐based screening experiment performed with OSM‐treated human dermal fibroblasts (HDF, not shown) indicated that OSM might induce the expression of a panel of ISG, including the RLR RIG‐I (gene: *DDX58*) and MDA5 (gene: *IFIH1*), as well as the transcription factor IRF1. To confirm these results, we stimulated HDF for different time periods with OSM and examined mRNA and protein expression by qRT‐PCR and Western Blot analysis. We found a strong and statistically significant increase in *IRF1*,* DDX58* and *IFIH1* mRNA levels as early response to OSM stimulation (Fig. 1A–C). The expression of *DDX58* and *IFIH1* mRNA peaked after 2–3 hrs of stimulation and declined thereafter, while the increase in *IRF1* mRNA expression preceded the helicase induction with a maximum after 1 hr of OSM stimulation. Similar results were obtained on protein level. IRF1 protein was present up to 3 hrs; RIG‐I and MDA5 protein expression peaked after 5 hrs stimulation and slowly returned to basal levels thereafter. Interestingly, besides IRF1, two other IRF essential for the antiviral response, namely IRF7 and IRF9, were found to be OSM target genes (Fig. 1D and E). Similar to *DDX58* and *IFIH1*, enhanced transcription of *IRF7* and *IRF9* mRNA in response to OSM was detectable with a maximum at 3 hrs of stimulation.

Taken together, our data demonstrate that OSM induces a transient expression of ISG in primary fibroblasts, characterized by an early and strong IRF1 response followed by the induction of RIG‐I, MDA5, IRF7 and IRF9.

### Expression of the LIFR is dispensable for the OSM‐induced expression of ISG

**Figure 2:**
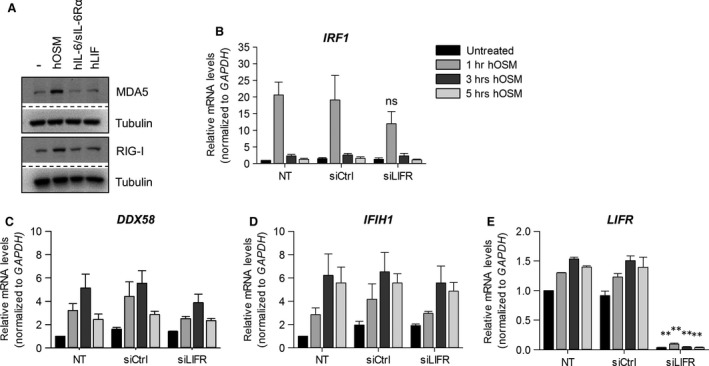
OSM mediates expression of ISG through the type II gp130/OSMR complex. **(A)** human dermal fibroblasts (HDF) were treated with 20 ng/ml hOSM, 20 ng/ml hIL‐6 and 500 ng/ml hsIL‐6Rα or 10 ng/ml hLIF for 5 hrs. Whole cellular extracts were analysed by SDS‐PAGE and Western blot for MDA5 and RIG‐I. Tubulin staining served as loading control. Blots shown are representative for three experiments. **(B–E)** HDF were incubated only with transfection reagent (NT) or transfected with control siRNA (siCtrl) or *LIFR* siRNA (siLIFR). At day two post‐transfection, cells were stimulated with 20 ng/ml hOSM for indicated time periods. Total RNA was prepared and subjected to reverse transcription. **(B)** *IRF1,* **(C)** *DDX58*,** (D)** *IFIH1* and **(E)** *LIFR* mRNA levels were analysed by qRT‐PCR. Relative mRNA levels were normalized to *GAPDH,* and fold changes were calculated relative to untreated, NT sample (set to 1). Shown are the means (*n* = 3) with S.E.M. Statistical significance was assessed by unpaired *t*‐test. **P ≤ 0.01, *versus* siCtrl.

Human fibroblasts express gp130, LIFR and OSMR. Therefore, they are not only responsive to OSM, but additionally to IL‐6 trans‐signalling 37 and LIF. To investigate whether the induction of RLR expression is a more general response of human fibroblasts to IL‐6‐type cytokines, we stimulated HDF either with LIF or with IL‐6 in combination with its soluble α‐receptor (sIL‐6Rα) for 5 hrs and analysed the protein expression of RIG‐I and MDA5 (Fig. 2A). While OSM induced the expression of RIG‐I and MDA5, neither LIF nor IL‐6/sIL‐6R stimulation enhanced RIG‐I and MDA5 protein expression in HDF (Fig. 2A). Accordingly, we hypothesized that the OSM‐mediated expression of RLR is mediated by the type II OSM receptor complex (gp130/OSMR) and independent of LIFR expression. For this reason, we transfected HDF with siRNA to LIFR and analysed the OSM‐induced *DDX58*,* IFIH1* and *IRF1* mRNA expression by qRT‐PCR. Efficient knock‐down of the LIFR, shown by a complete loss of the *LIFR* mRNA expression (Fig. 2E), did not significantly influence the OSM‐induced helicase and *IRF1* expression (Fig. 2B–D). Therefore, despite the fact that human OSM has equal affinity for both human LIFR and human OSMR, our data suggest that signalling through OSMR is sufficient to induce RIG‐I, MDA5 and IRF expression in HDF. However, a minor contribution of the type I receptor complex cannot be excluded.

### Helicase expression depends on STAT1 tyrosine and serine phosphorylation

**Figure 3:**
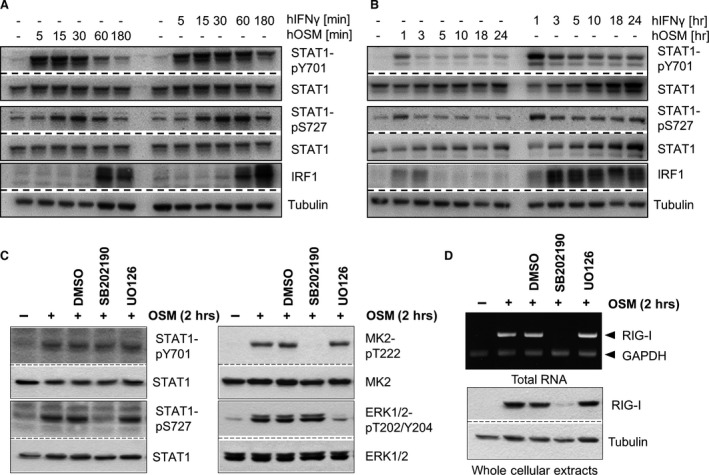
OSM‐induced serine phosphorylation of STAT1 is required for RIG‐I expression. **(A, B)** human dermal fibroblasts were treated with 20 ng/ml hOSM or 1000 U/ml IFN‐γ for the indicated time periods. Whole cellular extracts were analysed by Western blot for tyrosine/serine phosphorylation of STAT1, total STAT1 and IRF1. Tubulin staining served as loading control (*n* = 3). **(C, D)** HLF were pre‐treated with 10 μM SB202190, 10 μM U0126 or DMSO for 30 min. before being stimulated with 20 ng/ml OSM for 2 hrs. **(C)** Whole cellular extracts were analysed by Western blot for tyrosine/serine‐phosphorylated STAT1, threonine‐phosphorylated MK2 and threonine/tyrosine‐phosphorylated ERK1/2. Blots were stripped and reprobed with antibodies recognizing the proteins irrespective of their phosphorylation status. Blots shown are representative for two experiments. **(D)** upper panel: mRNA levels of RIG‐I and GAPDH were analysed from reverse‐transcribed total RNA (*n* = 3); lower panel: RIG‐I protein expression was analysed by Western blot using specific antibodies. Tubulin staining served as loading control. Blots shown are representative for three experiments.

To get a hint for the mechanism of OSM‐induced RLR expression, we compared the tyrosine and serine STAT1 phosphorylation upon stimulation with OSM or IFN‐γ, a known inducer of RIG‐I 8, 9, 38, 39. As expected, OSM and IFN‐γ induced a fast and strong tyrosine STAT1 phosphorylation (5 min.) followed by a slightly delayed serine STAT1 phosphorylation (15 min., Fig. 3A). While the IFN‐γ‐induced tyrosine and serine STAT1 phosphorylation was present up to 24 hrs (Fig. 3B), particularly the OSM‐induced STAT1 serine phosphorylation was more transient (Fig. 3A and B). Similarly, IRF1 protein expression peaked in response to OSM between 1 hr and 3 hrs after stimulation, while a clear increase in IRF1 protein was still detectable after 24 hrs of IFN‐γ stimulation (Fig. 3B). The transient OSM‐induced STAT1 phosphorylation correlated well with the transient increase in RIG‐I, MDA5 and IRF1 expression (Fig. 1). To further investigate whether the helicase and IRF expression relies indeed on dual‐phosphorylated STAT1, cultured fibroblasts were stimulated with OSM after pre‐treatment with well‐established pharmacological inhibitors against the p38 MAPK (SB202190) or MEK1 (U0126). Both kinases have been implicated in the serine phosphorylation of STAT1 40, 41.

As expected, none of the inhibitors prevented OSM‐mediated STAT1 tyrosine phosphorylation (Fig. 3C, left, top panel). However, the presence of SB202190 prevented OSM‐induced serine 727 phosphorylation of STAT1 (Fig. 3C, left, third panel). The analysis of MK2 and ERK1/2 phosphorylation (Fig. 3C, right) proved the specificity of the used inhibitors. Interestingly, despite an intact STAT1 tyrosine phosphorylation, the induction of RIG‐I mRNA and protein expression in response to OSM treatment is dependent on the activity of p38, but not MEK1/2 (Fig. 3D). This strongly suggested that STAT1 serine phosphorylation is a prerequisite for the subsequent initiation of RLR gene expression.

**Figure 4:**
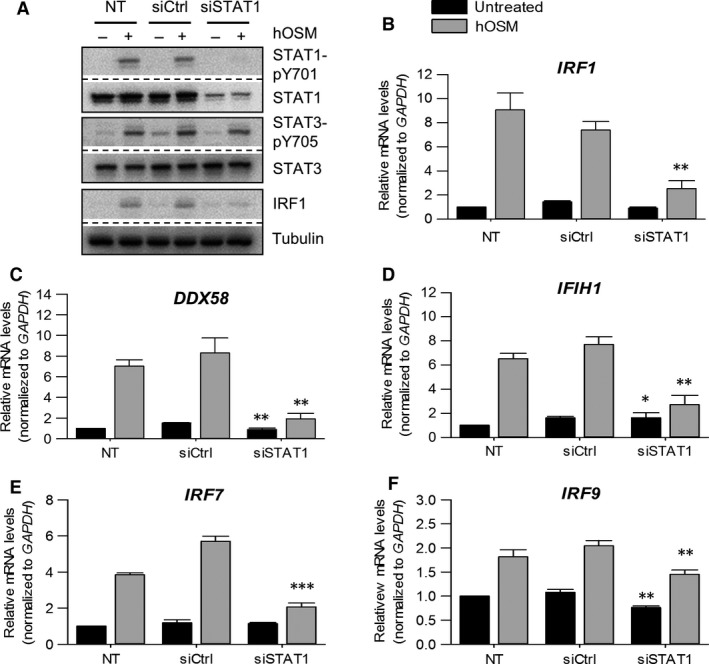
OSM‐induced DExD/H‐Box RNA helicase and IRF expression is STAT1 dependent. Human dermal fibroblasts were incubated only with transfection reagent (NT) or transfected with STAT1 siRNA (siSTAT1) or control siRNA (siCtrl). At day two post‐transfection, cells were stimulated with 20 ng/ml hOSM for one **(A)** or two **(B–F)** hrs. **(A)** Whole cellular extracts were prepared and phosphorylation of STAT1 and STAT3 as well as IRF1 expression were determined by Western blot analysis using specific antibodies. The blots were stripped and reprobed with antibodies either recognizing the proteins irrespective of their activation status or detecting tubulin as independent loading control (*n* = 3). Total RNA was prepared and subjected to reverse transcription. **(B)** *IRF1*,** (C)** *DDX58*,** (D)** *IFIH1*,** (E)** *IRF7* and **(F)** *IRF9* mRNA levels were analysed by qRT‐PCR. Relative mRNA levels were normalized to *GAPDH,* and fold changes were calculated relative to untreated, NT sample (set to 1). Shown are the means (*n* = 3) with S.E.M. Statistical significance was assessed by unpaired *t*‐test. **P ≤ 0.01 *versus* siCtrl.

To further strengthen the importance of STAT1 in the OSM‐induced RIG‐I, MDA5 and IRF expression, we used STAT1 SMART pool siRNA to specifically downregulate STAT1 expression in HDF. As shown in Figure 4A, STAT1 siRNA transfection strongly decreased STAT1 protein expression and thus prevented the OSM‐induced tyrosine STAT1 phosphorylation and IRF1 protein expression in HDF. The comparison with control siRNA‐treated cells revealed that transfection did not affect the OSM‐induced tyrosine STAT3 phosphorylation, and thus, IRF1 protein expression depends on OSM‐induced STAT1 signalling. Consequently, we also observed a significant reduction in *IRF1* mRNA levels in STAT1 siRNA‐transfected, OSM‐stimulated HDF (Fig. 4B). The same result was obtained for *DDX58*,* IFIH1*,* IRF7* and *IRF9* (Fig. 4C–F) mRNA levels.

In conclusion, the OSM‐mediated expression of RIG‐I, MDA5 and IRF1/7/9 in human fibroblasts depends on STAT1 tyrosine and serine phosphorylation.

### RNA interference‐mediated abrogation of STAT3 expression prolongs OSM‐induced STAT1 signalling and ISG expression

It was demonstrated that murine embryonic fibroblasts (MEF) from STAT3‐deficient mice display an enhanced RIG‐I and MDA5 expression upon viral infection 42 as well as an IFN‐γ‐like response after stimulation with IL‐6/sIL‐6Rα 43. Both, Wang *et al*. and Costa‐Pereira *et al*. provide evidence that STAT3 acts as a negative regulator either of the type I IFN or IL‐6 response, as viral infection and/or cytokine activation of STAT3‐deficient MEF resulted in enhanced/prolonged STAT1 phosphorylation and ISG expression.

**Figure 5:**
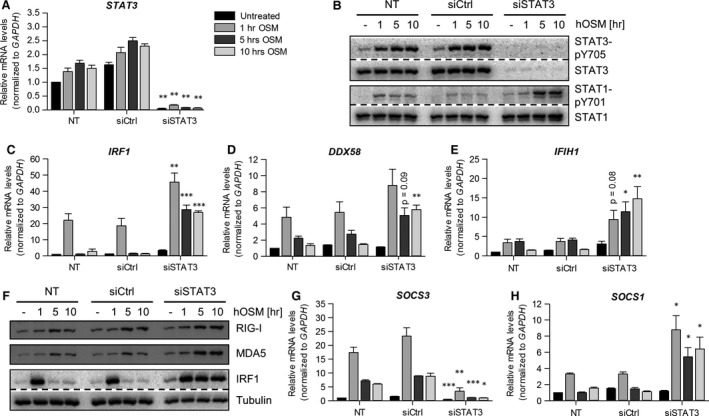
*STAT3* knock‐down prolongs OSM‐induced STAT1 phosphorylation and ISG expression. Human dermal fibroblasts were transfected with STAT3 siRNA (siSTAT3), control siRNA (siCtrl) or only treated with transfection reagent (NT). At day two post‐transfection, cells were stimulated with 20 ng/ml hOSM for indicated time period. Total RNA was prepared and subjected to reverse transcription. **(A)** *STAT3,* **(C)** *IRF1,* **(D)** *DDX58*,** (E)** *IFIH1,* **(G)** *SOCS3* and **(H)** *SOCS1* mRNA levels were analysed by qRT‐PCR. Relative mRNA levels were normalized to *GAPDH,* and fold changes were calculated relative to untreated, NT sample (set to 1). Shown are the means (*n* = 3) with S.E.M. Statistical significance was assessed by unpaired *t*‐test. *P ≤ 0.05, **P ≤ 0.01, ***P ≤ 0.001, *versus* siCtrl. **(B, F)** Whole cellular extracts were prepared, and tyrosine phosphorylation of STAT3 and STAT1 **(B)** as well as expression of RIG‐I, MDA5 and IRF1 **(F)** were determined by Western blot analysis using specific antibodies. The blots were stripped and reprobed with antibodies recognizing the proteins irrespective of their activation status. Tubulin was included as loading control. Blots shown are representative for three experiments.

OSM represents probably the strongest STAT3 activator within the family of IL‐6‐type cytokines, and STAT3 tyrosine phosphorylation in response to OSM is detectable for at least 24 hrs after OSM stimulation of HDF 44. Thus, we asked whether STAT3 was a key regulator of STAT1 signalling in OSM‐stimulated fibroblasts. As shown in Figure 5A and B, transfection of HDF with siRNA to STAT3 resulted in a complete loss of STAT3 mRNA and protein expression as well as STAT3 tyrosine phosphorylation. On the other hand, a marked increase in the level of tyrosine‐phosphorylated STAT1 was detectable after 5 hrs and 10 hrs of OSM stimulation in *STAT3* siRNA‐treated HDF (Fig. 5B). Additionally, levels of total STAT1 protein increased over time in OSM‐stimulated, *STAT3* siRNA‐transfected HDF but not in control siRNA‐transfected cells (Fig. 5B, 10 hrs stimulation).

To determine whether the increased OSM‐induced STAT1 phosphorylation results in an altered induction of ISG expression, we analysed mRNA and protein expression of RIG‐I, MDA5 and IRF1 in *STAT3* knock‐down and control cells. As shown in Figure 5C–F, abrogation of STAT3 expression resulted in an enhanced and significantly prolonged induction of *DDX58*,* IFIH1* and *IRF1* mRNA as well as RIG‐I, MDA5 and IRF1 protein expression. Taken together, these data demonstrate that STAT3 may directly or indirectly restrict the OSM‐induced STAT1‐dependent expression of RIG‐I, MDA5 and IRF1.

### SOCS3—main regulator of the OSM‐induced STAT signalling

One of the major factors limiting IL‐6‐type cytokine responses, including OSM, is the suppressor of cytokine signalling 3 (SOCS3) 45, 46, 47. As a direct target gene of STAT3, SOCS3 is considered to act as feedback inhibitor that shuts down cytokine‐induced signal transduction to prevent chronic activation of signalling pathways. Indeed, in STAT3 siRNA‐transfected HDF, no induction of *SOCS3* mRNA was detectable in response to OSM stimulation (Fig. 5G).

On the contrary, the STAT1‐dependent target gene *SOCS1* was induced more pronouncedly and for significantly longer periods of time in response to OSM in *STAT3* siRNA‐treated HDF (Fig. 5H). This corresponded nicely with the prolonged and enhanced STAT1 tyrosine phosphorylation in *STAT3* siRNA‐transfected HDF (Fig. 5B).

**Figure 6:**
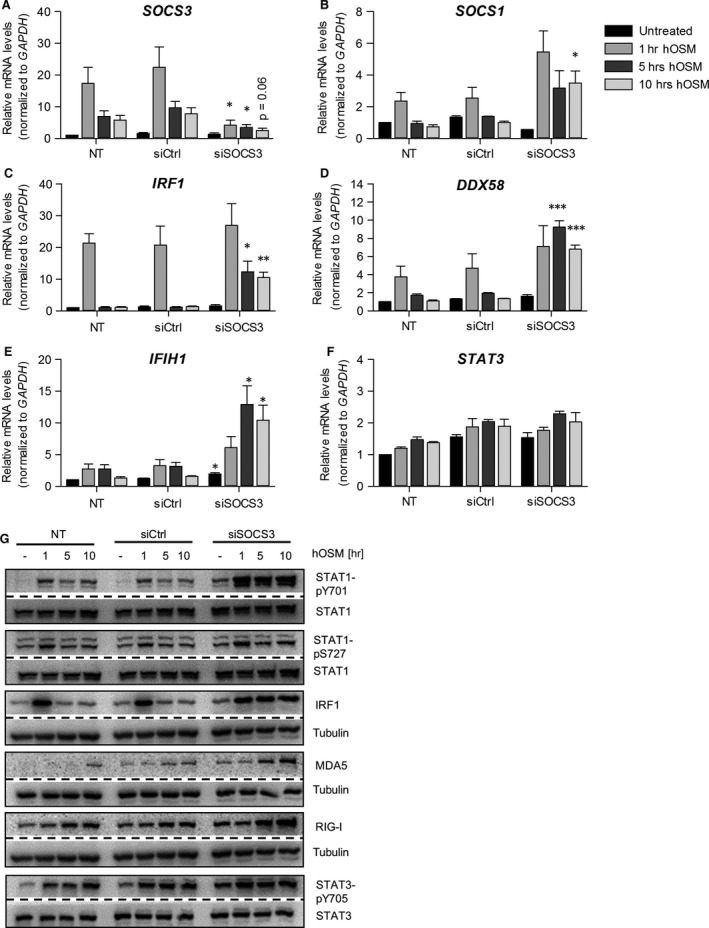
*SOCS3* knock‐down prolongs OSM‐induced ISG expression. Human dermal fibroblasts were transfected with *SOCS3* siRNA (siSOCS3), control siRNA (siCtrl) or only incubated with transfection reagent (NT). At day two post‐transfection, cells were stimulated with 20 ng/ml hOSM for indicated time periods. Total RNA was prepared and subjected to reverse transcription. **(A)** *SOCS3,* **(B)** *SOCS1*,** (C)** *IRF1*,** (D)** *DDX58,* **(E)** *IFIH1* and **(F)** *STAT3* mRNA levels were analysed by qRT‐PCR. Relative mRNA levels were normalized to *GAPDH,* and fold changes were calculated relative to untreated, NT sample (set to 1). Shown are the means (*n* = 3) with S.E.M. Statistical significance was assessed by unpaired *t*‐test. *P ≤ 0.05, **P ≤ 0.01, ***P ≤ 0.001, *versus* siCtrl. **(G)** Whole cellular extracts were prepared and phosphorylation of STAT1 and STAT3 and also expression of RIG‐I, MDA5 and IRF1 were determined by Western blot analysis using specific antibodies. The blots were stripped and reprobed with antibodies recognizing the proteins irrespective of their activation status. Tubulin was included as loading control. Blots shown are representative for three experiments.

To investigate whether SOCS3 might indeed not only be involved in shutdown of OSM‐mediated signalling, but rather in fine‐tuning the OSM‐mediated STAT signalling pathways, we next transfected cells with siRNA to *SOCS3*. As shown in Figure 6A, levels of OSM‐induced *SOCS3* mRNA were almost absent in cells treated with *SOCS3* siRNA compared with control siRNA‐transfected HDF. Consistent with the results obtained in HDF transfected with *STAT3* siRNA, STAT1 tyrosine phosphorylation (Fig. 6G, top panel) and *SOCS1* gene expression (Fig. 6B) were strongly increased in *SOCS3* siRNA‐transfected and OSM‐stimulated HDF. This correlated with a prolonged induction of ISG gene expression. *IRF1*,* DDX58* and *IFIH1* mRNA (Fig. 6C–E) and protein (Fig. 6G) were significantly stronger and longer detectable after OSM treatment of HDF transfected with *SOCS3* siRNA compared to control siRNA‐transfected cells. Intriguingly, despite the absence of SOCS3, *STAT3* mRNA levels (Fig. 6F) and STAT3 tyrosine phosphorylation (Fig. 6G, last panel) were only mildly enhanced in response to OSM. We therefore conclude that STAT3‐mediated SOCS3 induction affects the OSM‐induced STAT1 tyrosine and serine phosphorylation much more pronouncedly than the OSM‐induced STAT3 activation. Thereby, the initially induced STAT1 activation is rapidly turned off preventing prolonged activation of ISG in response to OSM.

Recent publications implicated that a number of additional proteins modify the STAT1/IRF‐dependent expression of RLR. These include the tyrosine phosphatase PTPN2 which has been shown to be involved in STAT1 dephosphorylation 48, the small ubiquitin‐like modifier (SUMO) 49 and the promyelocytic leukaemia protein (PML) 50, 51. To investigate whether these factors interfere with OSM‐mediated induction of *IRF1*,* DDX58* or *IFIH1*, HDF were transiently transfected with siRNA targeting each of these factors and changes in mRNA induction were evaluated in comparison with control siRNA‐transfected cells (Fig. [Link], [Link], [Link]). Despite efficient knock‐down of all targets, the transience in OSM‐mediated induction of ISG was unaffected. *PTPN2* knock‐down slightly prolonged *IRF1* induction (Fig. S1), which, however, did not significantly affect RLR expression and *SUMO1* knock‐down showed a trend towards slightly enhanced *DDX58* and *IFIH1* mRNA levels after 3 hrs of OSM stimulation (Fig. S2). Taken together, in comparison with STAT1, STAT3 and SOCS3, TC‐PTP and SUMO1 appear to play a minor role in OSM‐mediated expression of the analysed ISG, while PML is completely dispensable.

### Pre‐treatment with OSM enhances the IFN‐γ‐induced RLR expression and sensitizes HDF to double‐stranded RNA

**Figure 7:**
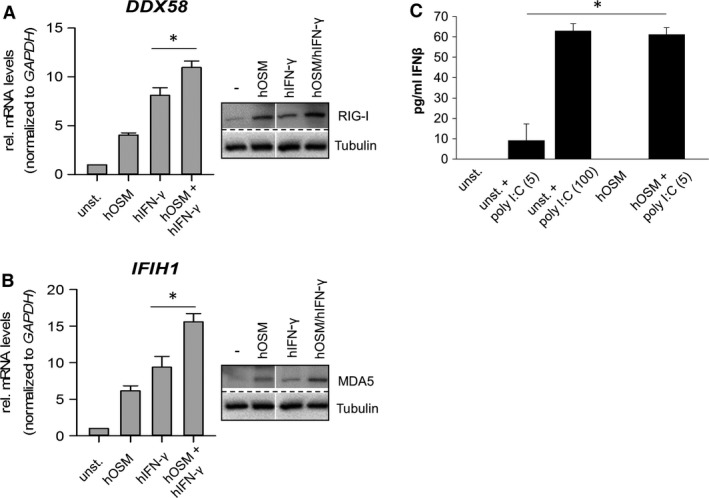
OSM and IFN‐γ additively induce DExD/H‐Box RNA helicase expression. **(A, B)** human dermal fibroblasts (HDF) were preincubated for two (RNA) or three hours (protein) with 20 ng/ml hOSM, followed by stimulation with 1000 U/ml IFN‐γ for 2 hrs. Total RNA was prepared and subjected to reverse transcription. **(A, left)** *DDX58* and **(B, left)** *IFIH1* mRNA levels were analysed by qRT‐PCR. Relative mRNA levels were normalized to *GAPDH,* and fold changes were calculated relative to unstimulated sample (set to 1). Shown are the means (*n* = 3) with S.E.M. Statistical significance was assessed by paired *t*‐test (*P ≤ 0.05). **(A, B right)** Whole cellular extracts were prepared and RIG‐I and MDA5 expression was determined by Western blot analysis using specific antibodies. Tubulin was included as loading control. Blots shown are representative for three experiments. **(C)** HDF were pre‐incubated for 2 hrs with 20 ng/ml OSM (columns 4,5) before they were left untreated (columns 1,4) or stimulated with 5 or 100 μg/ml poly I:C (columns 2,3,5) as indicated. Supernatants were collected and analysed by ELISA for the secretion of IFN‐β. Shown are means (*n* = 4) + S.E.M. Statistical significance was assessed by Mann–Whitney U‐test *P ≤ 0.05 (unst. + poly I:C (5) *versus* hOSM + poly I:C(5)).

To investigate whether OSM and IFN‐γ could indeed act together to increase the antiviral response in HDF, cells were pre‐treated with OSM followed by stimulation with IFN‐γ. The induction of *DDX58* as well as *IFIH1* mRNA expression was significantly stronger using a combination of OSM and IFN‐γ (Fig. 7A and B, left panels) which translated in a more pronounced RIG‐I and MDA5 protein expression when both cytokines were present (right panels). This suggests that OSM‐mediated signalling could cooperate with IFN‐γ to enhance the antiviral response.

A major task for the RNA helicases RIG‐I and MDA5 is the detection of double‐stranded RNA (dsRNA) in the cytoplasm of cells, which is indicative of a viral infection. As treatment of fibroblasts with OSM resulted in an enhanced expression of RLR, we postulated that cells should become more sensitive to the presence of dsRNA. A widely used model for mimicking viral dsRNA is stimulation of cells with poly I:C. We therefore incubated HDF with 5 or 100 μg/ml poly I:C, 20 ng/ml OSM or pre‐treated the cells with OSM for 2 hrs and subsequently stimulated with 5 μg/ml poly I:C. By itself, the lower concentration of poly I:C was insufficient to provoke a substantial expression and release of IFN‐β (Fig. 7C, 2^nd^ column). However, pre‐treatment of HDF with OSM allowed secretion of IFN‐β in response to 5 μg/ml poly I:C (Fig. 7C, 5^th^ column). Of note, the amount of IFN‐β produced upon pre‐stimulation of HDF with OSM was equivalent to that observed in response to 100 μg/ml poly I:C in unprimed cells (Fig. 7C, 3^rd^ column).

## Discussion

Initiation of the early immune response starts with the activation of pattern recognition receptors (PRRs), which bind to conserved molecular structures of pathogens known as pathogen‐associated molecular patterns (PAMPs). As host infection occurs *via* multiple routes, PRR are found at various cellular membranes (TLRs, C‐leptin receptors) as well as within the cytoplasm (RLR, NOD) 52, 53. RIG‐I‐like receptors (RLR) are cytoplasmic RNA helicases, which are expressed at low levels in resting cells, but their expression is strongly induced during viral infection by secretion of type I interferons 6, 54 as well as proinflammatory cytokines including IFN‐γ, IL‐1β and TNF 4, 9, 11, 12, 13, 38, 39, 55, 56. It has been demonstrated that RIG‐I is constitutively expressed in cells of the connective tissue including fibroblasts, epithelial and endothelial cells 1, 2, 3, 4, 5. Moreover, it has been shown that RIG‐I‐deficient MEF failed to generate IFN‐β upon viral infection, while virus‐infected dendritic cells from RIG‐I^−/−^ mice secrete large amounts of type I IFN 1, indicating that RIG‐I plays an important role in non‐professional immune cells.

In the current study, we demonstrate for the first time that the RLR RIG‐I and MDA5 as well as the transcription factors IRF1, IRF7 and IRF9 are strongly but transiently induced by OSM in HDF, whereas neither IL‐6 nor LIF augmented their expression. Interestingly, increased transcription was not observed in HepG2 hepatoma or U2OS osteosarcoma cells, which implicates that the response might be restricted to certain cell types. We provide evidence that the OSM‐induced expression depends on STAT1 phosphorylation, as helicase and also IRF1/IRF7/IRF9 expression were significantly inhibited in STAT1‐depleted cells. Of note, LPS‐mediated expression of RIG‐I and MDA5 was shown to be dependent on STAT2 expression and type I IFN signalling 57. However, we have no evidence that OSM leads to tyrosine phosphorylation of STAT2 and therefore exclude the involvement of STAT2 in OSM‐mediated ISG expression. Due to the observation that single Tyr701 STAT1 phosphorylation was not sufficient to induce RIG‐I expression, we suggest that the OSM‐induced expression depends on the simultaneously induced tyrosine and serine STAT1 phosphorylation, known to be required for maximal transcriptional activity of STATs 58, 59. Apparently, OSM‐mediated serine phosphorylation of STAT1 is dependent on p38 MAPK activity. In fibroblasts, OSM induced a Ser727 phosphorylation of STAT1 that was comparable in strength to IFN‐γ, while no Ser727 phosphorylation was detectable in SB202190 pre‐treated cells. The differential regulation/kinetics of *IRF1* and *DDX58/IFIH1* gene expression by OSM led us to assume that the IRF1 protein expression might be a prerequisite for induction of *DDX58/IFIH1* expression. In support of this hypothesis, previous findings indicate that mutation of a putative STAT1 binding site within the RIG‐I *(DDX58)* promoter region had no effect on promoter activity, while mutation or deletion of the IRF1 binding site markedly reduced the RIG‐I promoter activity 60. Additionally, a stronger promoter activity is observed in IRF1‐overexpressing cells, whereas *IRF1* depletion by siRNA completely inhibited RIG‐I induction 60, 61. A comparable IRF1‐dependent mechanism for MDA5 expression has not been demonstrated so far.

SOCS proteins are the key physiological regulators of the cytokine‐induced JAK/STAT pathway. Here, we demonstrate that the STAT3‐induced *SOCS3* is crucial to control STAT1 signalling in OSM‐treated HDF and therefore limits OSM‐induced ISG expression. In the absence of *STAT3* or *SOCS3*, OSM treatment induced an enhanced and prolonged STAT1 signalling and ISG expression similar to that observed in IL‐6‐treated STAT3^−/−^ MEF or SOCS3^−/−^ macrophages 43, 47, 62. Croker *et al*. 47 and Lang *et al*. 62 further provide evidence that although IL‐6 induced both SOCS1 and SOCS3, IL‐6 signalling is specifically controlled by induction of SOCS3, as SOCS1 deficiency did not affect IL‐6‐induced STAT1 or STAT3 phosphorylation. Given that OSM induced an increase in SOCS1 mRNA expression in si*STAT3‐* and si*SOCS3*‐treated HDF, our data provide strong evidence that SOCS3 is a specific regulator of OSM signalling as well. Thereby, our data strengthen recent findings for a role of SOCS3 in the negative regulation of OSM‐mediated signalling carried out in other cell types 45, 46, 63. Importantly, our findings emphasize that SOCS3 does not act as a general inhibitor of OSM‐induced signalling, as STAT3 tyrosine phosphorylation was not as strongly affected by *SOCS3* knock‐down compared to STAT1 phosphorylation. This indicates that SOCS3 action is essential to shape and specify the OSM signalling in a variety of cells types/tissues, particularly by modulating the ratio of STAT1 to STAT3 activation. This concept of opposing effects of STAT1 and STAT3 has been recognized in other cellular systems as well 64, 65, 66, 67.

Although IFN‐γ is a strong inducer of helicases 8, 9, 38, 39, we clearly show that OSM cooperated with IFN‐γ to enhance helicase expression in HDF. *DDX58* and *IFIH1* mRNA analysis as well as RIG‐I and MDA‐5 protein analysis revealed at least an additive effect in OSM/IFN‐γ‐stimulated HDF. Furthermore, we observed a beneficial effect of OSM pre‐incubation to sensitize double‐stranded RNA and allow subsequent type I IFN production. As we did not observe a significant upregulation of TLR3 expression in response to OSM (not shown), we postulate that poly I:C which was added to the cells was recognized by RLR. Indeed, recent publications indicate that extracellular poly I:C is not solely recognized by TLR3 upon endocytosis, but by yet unknown mechanisms detected through MDA5 68, 69, 70. Of note, cytopathic assays carried out with encephalomyocarditis virus‐infected HDF indicated that OSM‐induced RLR expression was by itself insufficient to protect the cells from virus‐mediated cell death (data not shown). Taken together, we demonstrate that OSM induces the expression of early antiviral genes in fibroblasts including IRF1/7/9 as well as the RNA helicases RIG‐I and MDA5 and this allows a more rapid response to double‐stranded RNA. Consequently, in cooperation with type II IFN, OSM appears to enhance the antiviral response. Thereby, our data extend recent findings which showed a cross‐talk of OSM and IFNs in the protection from hepatitis C virus infection 19, 20, 21 by elucidating a novel, yet unaddressed mechanism of OSM‐mediated immunomodulatory activities.

## Author contributions

SH, CM and APCP performed research experiments and analysed the data; SH, CH, APCP and HMH designed the research study; SH, CM and HMH wrote the manuscript.

## Conflict of interest

The authors confirm that there are no conflict of interests.

## Supporting information


**Figure S1** PTPN2 knock‐down slightly prolongs the OSM‐induced ISG induction.

**Figure S2** SUMOylation appears to slightly decrease the OSM‐induced RLR induciton.

**Figure S3** PML knock‐down does not affect OSM‐induced ISG induction.

## References

[1] Kato H , Sato S , Yoneyama M , *et al* Cell type‐specific involvement of RIG‐I in antiviral response. Immunity. 2005; 23: 19–28. 16039576 10.1016/j.immuni.2005.04.010

[2] Ohta K , Fukui A , Shigeishi H , *et al* Expression and function of RIG‐I in oral keratinocytes and fibroblasts. Cell Physiol Biochem. 2014; 34: 1556–65. 25359319 10.1159/000366359

[3] Berghall H , Siren J , Sarkar D , *et al* The interferon‐inducible RNA helicase, mda‐5, is involved in measles virus‐induced expression of antiviral cytokines. Microbes Infect. 2006; 8: 2138–44. 16782388 10.1016/j.micinf.2006.04.005

[4] Matikainen S , Siren J , Tissari J , *et al* Tumor necrosis factor alpha enhances influenza A virus‐induced expression of antiviral cytokines by activating RIG‐I gene expression. J Virol. 2006; 80: 3515–22. 16537619 10.1128/JVI.80.7.3515-3522.2006 PMC1440408

[5] Kubota K , Sakaki H , Imaizumi T , *et al* Retinoic acid‐inducible gene‐I is induced in gingival fibroblasts by lipopolysaccharide or poly IC: possible roles in interleukin‐1 beta, ‐6 and ‐8 expression. Oral Microbiol Immunol. 2006; 21: 399–406. 17064399 10.1111/j.1399-302X.2006.00326.x

[6] Kang DC , Gopalkrishnan RV , Lin L , *et al* Expression analysis and genomic characterization of human melanoma differentiation associated gene‐5, mda‐5: a novel type I interferon‐responsive apoptosis‐inducing gene. Oncogene. 2004; 23: 1789–800. 14676839 10.1038/sj.onc.1207300

[7] Imaizumi T , Aratani S , Nakajima T , *et al* Retinoic acid‐inducible gene‐I is induced in endothelial cells by LPS and regulates expression of COX‐2. Biochem Biophys Res Commun. 2002; 292: 274–9. 11890704 10.1006/bbrc.2002.6650

[8] Imaizumi T , Yagihashi N , Hatakeyama M , *et al* Expression of retinoic acid‐inducible gene‐I in vascular smooth muscle cells stimulated with interferon‐gamma. Life Sci. 2004; 75: 1171–80. 15219805 10.1016/j.lfs.2004.01.030

[9] Kitamura H , Matsuzaki Y , Kimura K , *et al* Cytokine modulation of retinoic acid‐inducible gene‐I (RIG‐I) expression in human epidermal keratinocytes. J Dermatol Sci. 2007; 45: 127–34. 17182220 10.1016/j.jdermsci.2006.11.003

[10] Imaizumi T , Hatakeyama M , Yamashita K , *et al* Interferon‐gamma induces retinoic acid‐inducible gene‐I in endothelial cells. Endothelium. 2004; 11: 169–73. 15370293 10.1080/10623320490512156

[11] Imaizumi T , Kumagai M , Taima K , *et al* Involvement of retinoic acid‐inducible gene‐I in the IFN‐{gamma}/STAT1 signalling pathway in BEAS‐2B cells. Eur Respir J. 2005; 25: 1077–83. 15929965 10.1183/09031936.05.00102104

[12] Matsumiya T , Prescott SM , Stafforini DM . IFN‐epsilon mediates TNF‐alpha‐Induced STAT1 phosphorylation and induction of retinoic acid‐inducible gene‐i in human cervical cancer cells. J Immunol. 2007; 179: 4542–9. 17878351 10.4049/jimmunol.179.7.4542

[13] Sakaki H , Imaizumi T , Matsumiya T , *et al* Retinoic acid‐inducible gene‐I is induced by interleukin‐1 beta in cultured human gingival fibroblasts. Oral Microbiol Immunol. 2005; 20: 47–50. 15612946 10.1111/j.1399-302X.2005.00181.x

[14] Hirano T , Matsuda T , Hosoi K , *et al* Absence of antiviral activity in recombinant B cell stimulatory factor 2 (BSF‐2). Immunol Lett. 1988; 17: 41–5. 2832321 10.1016/0165-2478(88)90099-5

[15] Reis LFL , Le J , Hirano T , *et al* Antiviral action of tumor necrosis factor in human‐fibroblasts is not mediated by B‐cell stimulatory factor‐II IFN‐beta‐2, and is inhibited by specific antibodies to IFN‐beta. J Immunol. 1988; 140: 1566–70. 3279118

[16] Lauder SN , Jones E , Smart K , *et al* Interleukin‐6 limits influenza‐induced inflammation and protects against fatal lung pathology. Eur J Immunol. 2013; 43: 2613–25. 23857287 10.1002/eji.201243018 PMC3886386

[17] Dienz O , Rud JG , Eaton SM , *et al* Essential role of IL‐6 in protection against H1N1 influenza virus by promoting neutrophil survival in the lung. Mucosal Immunol. 2012; 5: 258–66. 22294047 10.1038/mi.2012.2 PMC3328598

[18] Gazel A , Rosdy M , Bertin B , *et al* A characteristic subset of psoriasis‐associated genes is induced by oncostatin‐m in reconstituted epidermis. J Invest Dermatol. 2006; 126: 2647–57. 16917497 10.1038/sj.jid.5700461

[19] Ikeda M , Mori K , Ariumi Y , *et al* Oncostatin M synergistically inhibits HCV RNA replication in combination with interferon‐alpha. FEBS Lett. 2009; 583: 1434–8. 19332062 10.1016/j.febslet.2009.03.054

[20] Larrea E , Aldabe R , Gonzalez I , *et al* Oncostatin M enhances the antiviral effects of type I interferon and activates immunostimulatory functions in liver epithelial cells. J Virol. 2009; 83: 3298–311. 19158240 10.1128/JVI.02167-08 PMC2655580

[21] Larrea E , Echeverria I , Riezu‐Boj JI , *et al* Characterization of the CD40L/Oncostatin M/Oncostatin M receptor axis as an antiviral and immunostimulatory system disrupted in chronic HCV infection. J Hepatol. 2014; 60: 482–9. 24418171 10.1016/j.jhep.2013.10.016

[22] Verhoeckx KC , Doornbos RP , Witkamp RF , *et al* Beta‐adrenergic receptor agonists induce the release of granulocyte chemotactic protein‐2, oncostatin M, and vascular endothelial growth factor from macrophages. Int Immunopharmacol. 2006; 6: 1–7. 16332507 10.1016/j.intimp.2005.05.013

[23] Malik N , Kallestad JC , Gunderson NL , *et al* Molecular cloning, sequence analysis, and functional expression of a novel growth regulator, oncostatin M. Mol Cell Biol. 1989; 9: 2847–53. 2779549 10.1128/mcb.9.7.2847-2853.1989 PMC362750

[24] Brown TJ , Lioubin MN , Marquardt H . Purification and characterization of cytostatic lymphokines produced by activated human T lymphocytes. Synergistic antiproliferative activity of transforming growth factor beta 1, interferon‐gamma, and oncostatin M for human melanoma cells. J Immunol. 1987; 139: 2977–83. 3117884

[25] Grenier A , Combaux D , Chastre J , *et al* Oncostatin M production by blood and alveolar neutrophils during acute lung injury. Lab Invest. 2001; 81: 133–41. 11232634 10.1038/labinvest.3780220

[26] Hurst SM , McLoughlin RM , Monslow J , *et al* Secretion of oncostatin M by infiltrating neutrophils: regulation of IL‐6 and chemokine expression in human mesothelial cells. J Immunol. 2002; 169: 5244–51. 12391243 10.4049/jimmunol.169.9.5244

[27] Suda T , Chida K , Todate A , *et al* Oncostatin M production by human dendritic cells in response to bacterial products. Cytokine. 2002; 17: 335–40. 12061841 10.1006/cyto.2002.1023

[28] Zarling JM , Shoyab M , Marquardt H , *et al* Oncostatin M: a growth regulator produced by differentiated histiocytic lymphoma cells. Proc Natl Acad Sci U S A. 1986; 83: 9739–43. 3540948 10.1073/pnas.83.24.9739 PMC387216

[29] Gearing DP , Comeau MR , Friend DJ , *et al* The IL‐6 signal transducer, gp130: an oncostatin M receptor and affinity converter for the LIF receptor. Science. 1992; 255: 1434–7. 1542794 10.1126/science.1542794

[30] Mosley B , De Imus C , Friend D , *et al* Dual oncostatin M (OSM) receptors. Cloning and characterization of an alternative signaling subunit conferring OSM‐specific receptor activation. J Biol Chem. 1996; 271: 32635–43. 8999038 10.1074/jbc.271.51.32635

[31] Tanaka M , Hirabayashi Y , Sekiguchi T , *et al* Targeted disruption of oncostatin M receptor results in altered hematopoiesis. Blood. 2003; 102: 3154–62. 12855584 10.1182/blood-2003-02-0367

[32] Hermanns HM . Oncostatin M and interleukin‐31: cytokines, receptors, signal transduction and physiology. Cytokine Growth Factor Rev. 2015; 26: 545–58. 26198770 10.1016/j.cytogfr.2015.07.006

[33] Hausmann M , Rogler G . Immune‐non immune networks in intestinal inflammation. Curr Drug Targets. 2008; 9: 388–94. 18473767 10.2174/138945008784221152

[34] Weiergraber O , Hemmann U , Kuster A , *et al* Soluble human interleukin‐6 receptor ‐ expression in insect cells, purification and characterization. Eur J Biochem. 1995; 234: 661–9. 8536717 10.1111/j.1432-1033.1995.661_b.x

[35] Hermanns HM , Radtke S , Schaper F , *et al* Non‐redundant signal transduction of interleukin‐6‐type cytokines ‐ The adapter protein Shc is specifically recruited to the oncostatin M receptor. J Biol Chem. 2000; 275: 40742–8. 11016927 10.1074/jbc.M005408200

[36] Pfaffl MW . A new mathematical model for relative quantification in real‐time RT‐PCR. Nucleic Acids Res. 2001; 29: e45. 11328886 10.1093/nar/29.9.e45 PMC55695

[37] Rose‐John S . IL‐6 trans‐signaling *via* the soluble IL‐6 receptor: importance for the pro‐inflammatory activities of IL‐6. Int J Biol Sci. 2012; 8: 1237–47. 23136552 10.7150/ijbs.4989 PMC3491447

[38] Yuzawa E , Imaizumi T , Matsumiya T , *et al* Retinoic acid‐inducible gene‐I is induced by interferon‐gamma and regulates CXCL11 expression in HeLa cells. Life Sci. 2008; 82: 670–5. 18258269 10.1016/j.lfs.2007.12.025

[39] Cui XF , Imaizumi T , Yoshida H , *et al* Retinoic acid‐inducible gene‐I is induced by interferon‐gamma and regulates the expression of interferon‐gamma stimulated gene 15 in MCF‐7 cells. Biochem Cell Biol. 2004; 82: 401–5. 15181474 10.1139/o04-041

[40] Goh KC , Haque SJ , Williams BR . p38 MAP kinase is required for STAT1 serine phosphorylation and transcriptional activation induced by interferons. EMBO J. 1999; 18: 5601–8. 10523304 10.1093/emboj/18.20.5601 PMC1171628

[41] Song JH , So EY , Lee CE . Increased serine phosphorylation and activation of STAT1 by oncogenic Ras transfection. Mol Cells. 2002; 13: 322–6. 12018856

[42] Wang WB , Levy DE , Lee CK . STAT3 negatively regulates Type I IFN‐mediated antiviral response. J Immunol. 2011; 187: 2578–85. 21810606 10.4049/jimmunol.1004128

[43] Costa‐Pereira AP , Tininini S , Strobl B , *et al* Mutational switch of an IL‐6 response to an interferon‐gamma‐like response. Proc Natl Acad Sci U S A. 2002; 99: 8043–7. 12060750 10.1073/pnas.122236099 PMC123017

[44] Dreuw A , Hermanns HM , Heise R , *et al* Interleukin‐6‐type cytokines upregulate expression of multidrug resistance‐associated proteins in NHEK and dermal fibroblasts. J Invest Dermatol. 2005; 124: 28–37. 15654950 10.1111/j.0022-202X.2004.23499.x

[45] Magrangeas F , Boisteau O , Denis S , *et al* Negative regulation of onconstatin M signaling by suppressor of cytokine signaling (SOCS‐3). Eur Cytokine Netw. 2001; 12: 309–15. 11399520

[46] Stross C , Radtke S , Clahsen T , *et al* Oncostatin M receptor‐mediated signal transduction is negatively regulated by SOCS3 through a receptor tyrosine‐independent mechanism. J Biol Chem. 2006; 281: 8458–68. 16459330 10.1074/jbc.M511212200

[47] Croker BA , Krebs DL , Zhang JG , *et al* SOCS3 negatively regulates IL‐6 signaling *in vivo* . Nat Immunol. 2003; 4: 540–5. 12754505 10.1038/ni931

[48] ten Hoeve J , de Jesus Ibarra‐Sanchez M , Fu Y , *et al* Identification of a nuclear Stat1 protein tyrosine phosphatase. Mol Cell Biol. 2002; 22: 5662–8. 12138178 10.1128/MCB.22.16.5662-5668.2002 PMC133976

[49] Kubota T , Matsuoka M , Chang TH , *et al* Virus infection triggers SUMOylation of IRF3 and IRF7, leading to the negative regulation of type I interferon gene expression. J Biol Chem. 2008; 283: 25660–70. 18635538 10.1074/jbc.M804479200 PMC2533075

[50] Choi YH , Bernardi R , Pandolfi PP , *et al* The promyelocytic leukemia protein functions as a negative regulator of IFN‐gamma signaling. Proc Natl Acad Sci U S A. 2006; 103: 18715–20. 17121994 10.1073/pnas.0604800103 PMC1693728

[51] Kim YE , Ahn JH . Positive role of promyelocytic leukemia protein in type I interferon response and its regulation by human cytomegalovirus. PLoS Pathog. 2015; 11: e1004785. 25812002 10.1371/journal.ppat.1004785 PMC4374831

[52] Kawai T , Akira S . Toll‐like receptors and their crosstalk with other innate receptors in infection and immunity. Immunity. 2011; 34: 637–50. 21616434 10.1016/j.immuni.2011.05.006

[53] Kumar H , Kawai T , Akira S . Pathogen recognition by the innate immune system. Int Rev Immunol. 2011; 30: 16–34. 21235323 10.3109/08830185.2010.529976

[54] Loo YM , Gale M Jr . Immune signaling by RIG‐I‐like receptors. Immunity. 2011; 34: 680–92. 21616437 10.1016/j.immuni.2011.05.003 PMC3177755

[55] Imaizumi T , Tanaka H , Tajima A , *et al* Retinoic acid‐inducible gene‐I (RIG‐I) is induced by IFN‐gamma in human mesangial cells in culture: possible involvement of RIG‐I in the inflammation in lupus nephritis. Lupus. 2010; 19: 830–6. 20167631 10.1177/0961203309360540

[56] Imaizumi T , Matsumiya T , Yoshida H , *et al* Tumor‐necrosis factor‐alpha induces retinoic acid‐inducible gene‐I in rheumatoid fibroblast‐like synoviocytes. Immunol Lett. 2009; 122: 89–93. 19126414 10.1016/j.imlet.2008.12.005

[57] Sheikh F , Dickensheets H , Gamero AM , *et al* An essential role for IFN‐beta in the induction of IFN‐stimulated gene expression by LPS in macrophages. J Leukoc Biol. 2014; 96: 591–600. 25024400 10.1189/jlb.2A0414-191R PMC4163629

[58] Wen Z , Zhong Z , Darnell JE Jr . Maximal activation of transcription by Stat1 and Stat3 requires both tyrosine and serine phosphorylation. Cell. 1995; 82: 241–50. 7543024 10.1016/0092-8674(95)90311-9

[59] Zhu X , Wen Z , Xu LZ , *et al* Stat1 serine phosphorylation occurs independently of tyrosine phosphorylation and requires an activated Jak2 kinase. Mol Cell Biol. 1997; 17: 6618–23. 9343425 10.1128/mcb.17.11.6618 PMC232515

[60] Wang F , Xia W , Liu F , *et al* Interferon regulator factor 1/retinoic inducible gene I (IRF1/RIG‐I) axis mediates 25‐hydroxycholesterol‐induced interleukin‐8 production in atherosclerosis. Cardiovasc Res. 2012; 93: 190–9. 21979142 10.1093/cvr/cvr260

[61] Su ZZ , Sarkar D , Emdad L , *et al* Central role of interferon regulatory factor‐1 (IRF‐1) in controlling retinoic acid inducible gene‐I (RIG‐I) expression. J Cell Physiol. 2007; 213: 502–10. 17516545 10.1002/jcp.21128

[62] Lang R , Pauleau AL , Parganas E , *et al* SOCS3 regulates the plasticity of gp130 signaling. Nat Immunol. 2003; 4: 546–50. 12754506 10.1038/ni932

[63] Ehlting C , Bohmer O , Hahnel MJ , *et al* Oncostatin M regulates SOCS3 mRNA stability *via* the MEK‐ERK1/2‐pathway independent of p38(MAPK)/MK2. Cell Signal. 2015; 27: 555–67. 25562430 10.1016/j.cellsig.2014.12.016

[64] Regis G , Pensa S , Boselli D , *et al* Ups and downs: the STAT1:sTAT3 seesaw of Interferon and gp130 receptor signalling. Semin Cell Dev Biol. 2008; 19: 351–9. 18620071 10.1016/j.semcdb.2008.06.004

[65] Friedrich K , Dolznig H , Han X , *et al* Steering of carcinoma progression by the YIN/YANG interaction of STAT1/STAT3. Biosci Trends. 2017; 11: 1–8. 28154246 10.5582/bst.2016.01250

[66] Grisouard J , Shimizu T , Duek A , *et al* Deletion of Stat3 in hematopoietic cells enhances thrombocytosis and shortens survival in a JAK2‐V617F mouse model of MPN. Blood. 2015; 125: 2131–40. 25595737 10.1182/blood-2014-08-594572

[67] Harwardt T , Lukas S , Zenger M , *et al* Human cytomegalovirus immediate‐early 1 protein rewires upstream STAT3 to downstream STAT1 signaling switching an IL6‐type to an IFNgamma‐like response. PLoS Pathog. 2016; 12: e1005748. 27387064 10.1371/journal.ppat.1005748 PMC4936752

[68] Chiang HS , Zhao Y , Song JH , *et al* GEF‐H1 controls microtubule‐dependent sensing of nucleic acids for antiviral host defenses. Nat Immunol. 2014; 15: 63–71. 24270516 10.1038/ni.2766 PMC4066330

[69] Ishii KJ , Koyama S , Nakagawa A , *et al* Host innate immune receptors and beyond: making sense of microbial infections. Cell Host Microbe. 2008; 3: 352–63. 18541212 10.1016/j.chom.2008.05.003

[70] McCartney S , Vermi W , Gilfillan S , *et al* Distinct and complementary functions of MDA5 and TLR3 in poly(I:c)‐mediated activation of mouse NK cells. J Exp Med. 2009; 206: 2967–76. 19995959 10.1084/jem.20091181 PMC2806445

