# Supplementary figures and images for: Oncostatin M induces RIG‐I and MDA5 expression and enhances the double‐stranded RNA response in fibroblasts
Source: J Cell Mol Med. 2017 May 30;21(11):3087–99. doi: 10.1111/jcmm.13221 (PMC5661242; doi:10.1111/jcmm.13221)

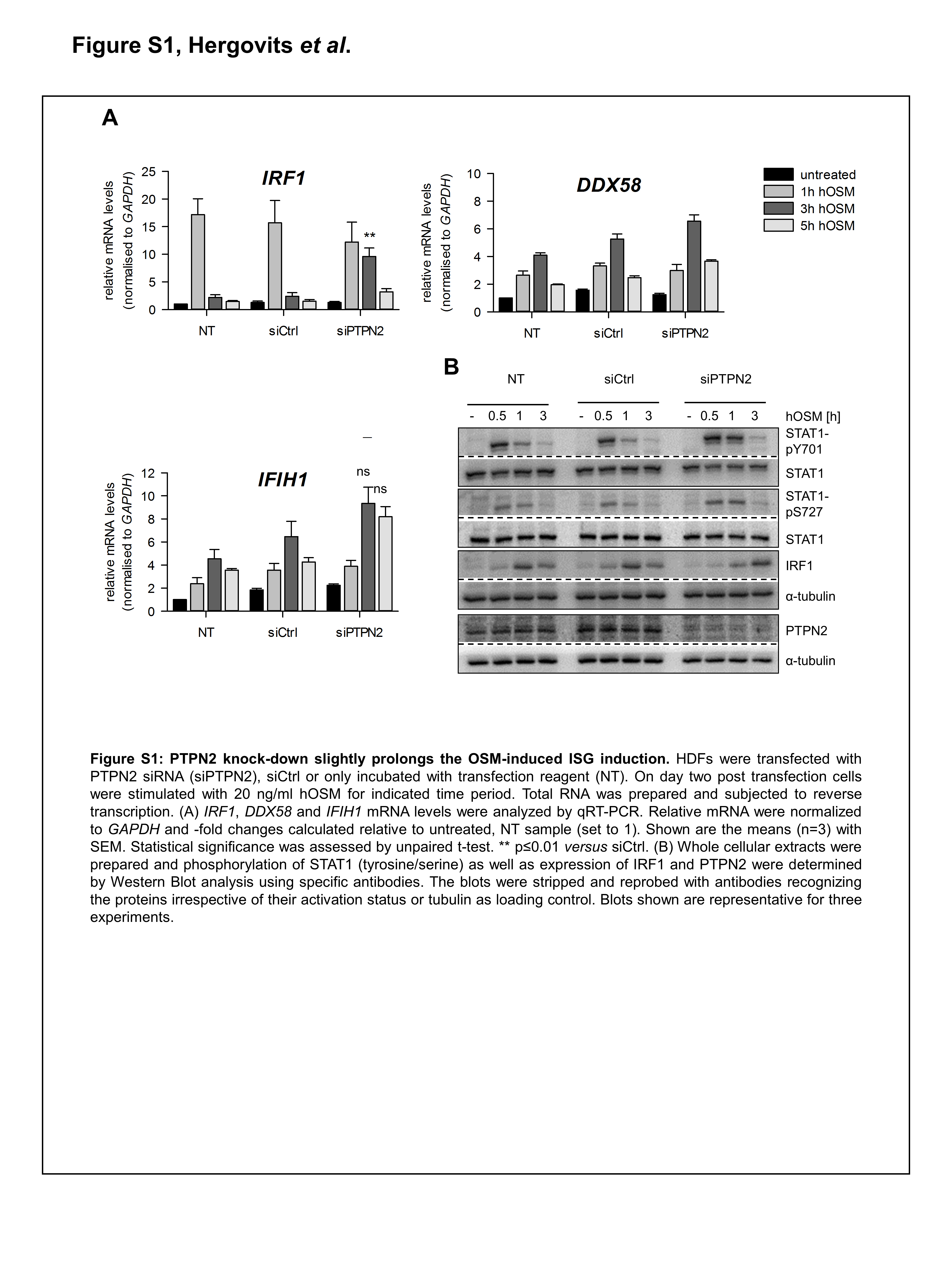

Supplement: Supplementary file 1 — Figure S1 PTPN2 knock‐down slightly prolongs the OSM‐induced ISG induction. [file JCMM-21-3087-s001.TIF]

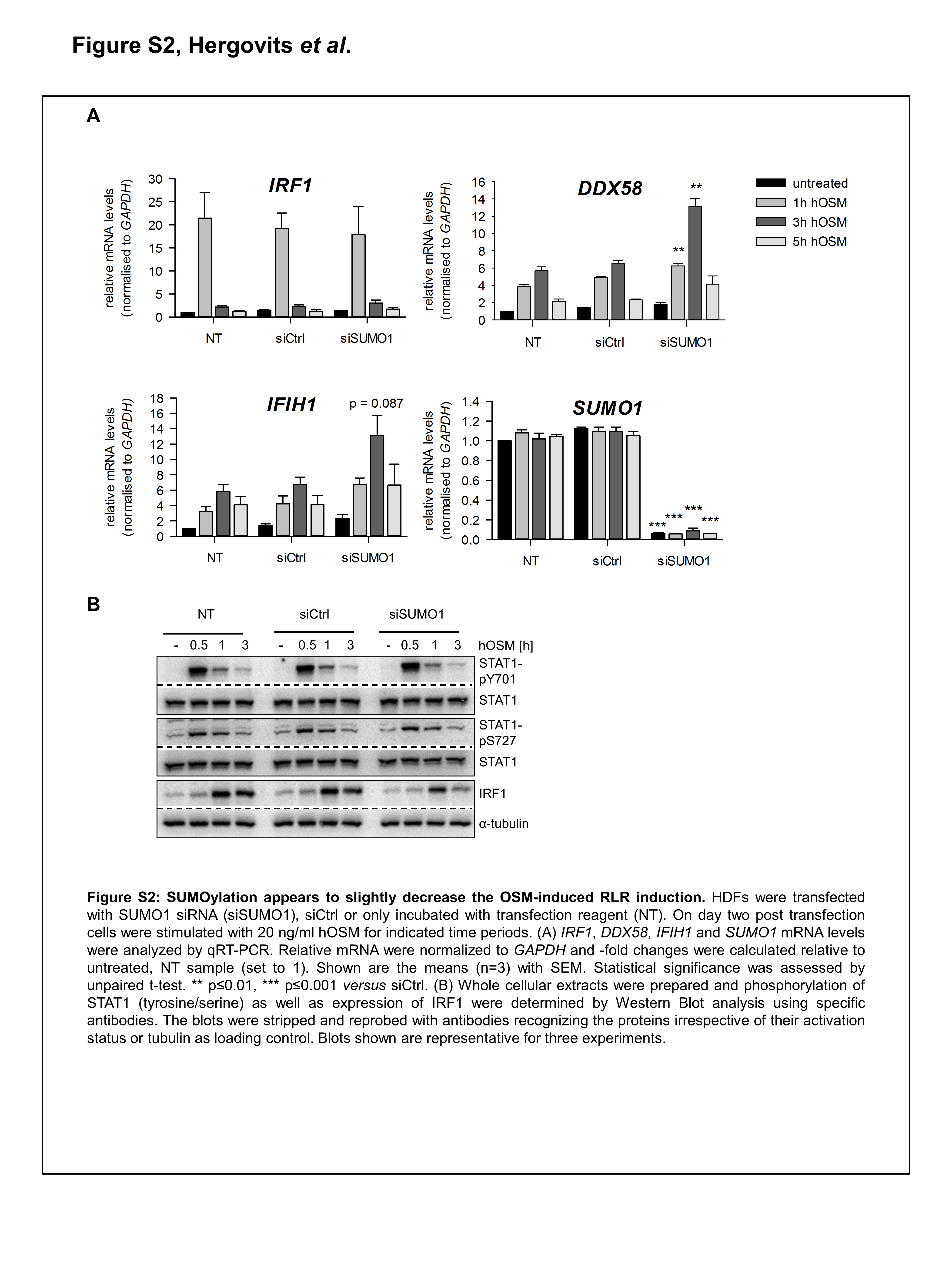

Supplement: Supplementary file 2 — Figure S2 SUMOylation appears to slightly decrease the OSM‐induced RLR induciton. [file JCMM-21-3087-s002.TIF]

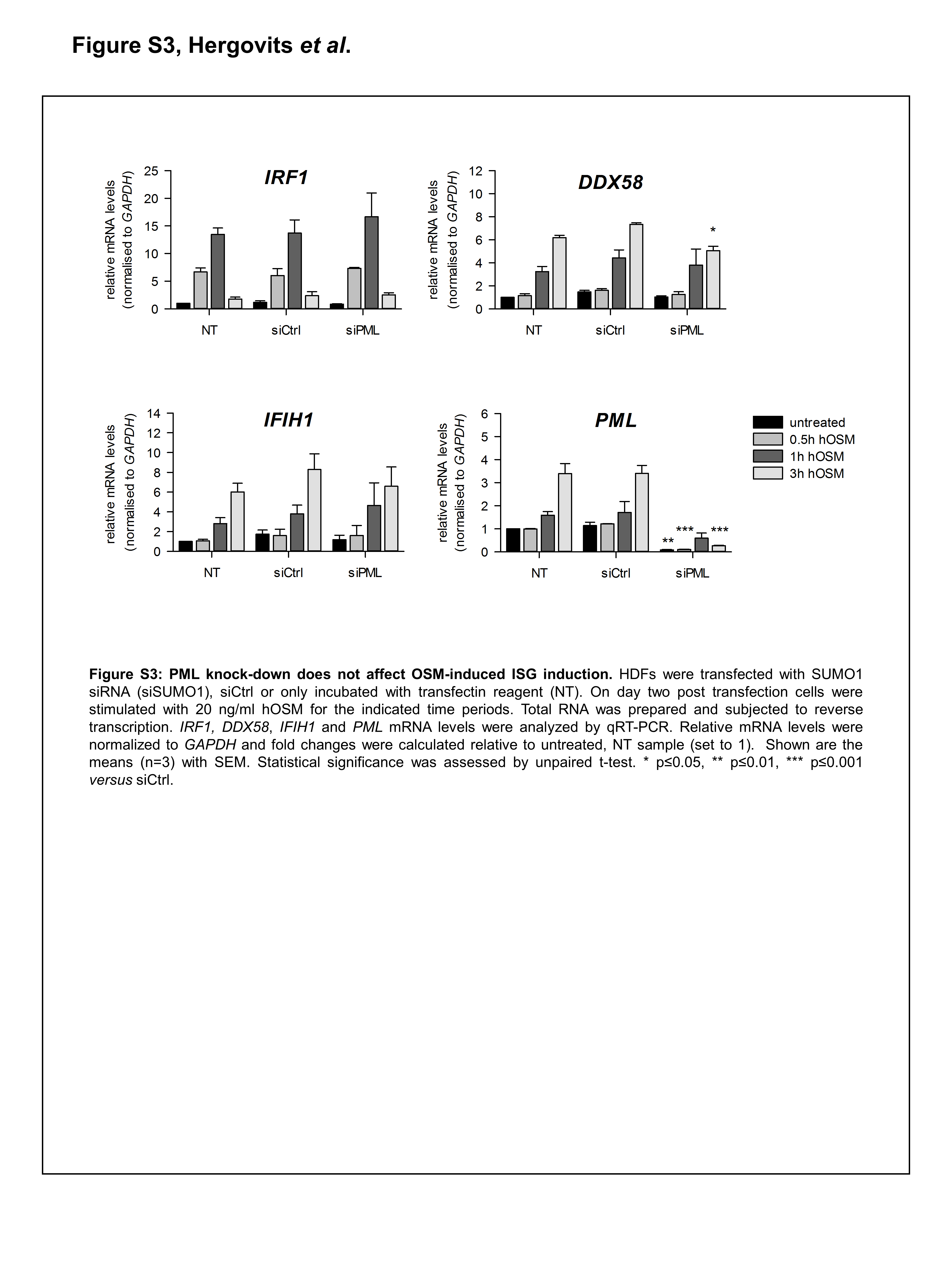

Supplement: Supplementary file 3 — Figure S3 PML knock‐down does not affect OSM‐induced ISG induction. [file JCMM-21-3087-s003.TIF]
